# Safety of Monoclonal Antibodies in Children Affected by SARS-CoV-2 Infection

**DOI:** 10.3390/children9030369

**Published:** 2022-03-07

**Authors:** Lorenza Romani, Francesca Ippolita Calò Carducci, Sara Chiurchiù, Laura Cursi, Maia De Luca, Martina Di Giuseppe, Andrzej Krzysztofiak, Laura Lancella, Paolo Palma, Leonardo Vallesi, Tiziana Corsetti, Andrea Campana, Emanuele Nicastri, Paolo Rossi, Stefania Bernardi

**Affiliations:** 1 Immunology and Infectious Disease Unit, Bambino Gesù Children’s Hospital, IRCCS, 00165 Rome, Italy; fcalocarducci@hotmal.com (F.I.C.C.); sarachiurchiu@gmail.com (S.C.); laura.cursi@opbg.net (L.C.); maia.deluca@opbg.net (M.D.L.); martina.digiuseppe@opbg.net (M.D.G.); andrzej.krzysztofiak@opbg.net (A.K.); laura.lancella@opbg.net (L.L.); stefania.bernardi@opbg.net (S.B.); 2 Research Unit of Congenital and Perinatal Infections, Bambino Gesù Children’s Hospital, IRCCS, 00165 Rome, Italy; paolo.palma@opbg.net; 3 Chair of Pediatrics, Department of Systems Medicine, University of Rome “Tor Vergata”, 00133 Rome, Italy; paolo.rossi@opbg.net; 4 Hospital Pharmacy Unit, Bambino Gesù Children’s Hospital, IRCCS, 00165 Rome, Italy; leonardo.vallesi@opbg.net (L.V.); tiziana.corsetti@opbg.net (T.C.); 5 Department of Pediatrics, Bambino Gesù Children’s Hospital, IRCCS, 00165 Rome, Italy; andrea.campana@opbg.net; 6 National Institute for Infectious Diseases, Lazzaro Spallanzani, IRCCS, 00149 Rome, Italy; emanuele.nicastri@opbg.net; 7 Academic Department of Pediatrics, Bambino Gesù Children’s Hospital, IRCCS, 00165 Rome, Italy

**Keywords:** COVID-19, monoclonal antibody, children

## Abstract

Monoclonal antibody therapies for COVID-19 have been frequently used in adults, whereas there are little data regarding the safety or efficacy of monoclonal antibody treatments in pediatric patients affected by COVID-19. We report our experience in the administration of mAb as a treatment for SARS-CoV-2 infection in children aged from 24 days to 18 years old.

## 1. Introduction

Severe acute respiratory syndrome (SARS-CoV-2) was first recognized in Wuhan in December 2019 and then spread rapidly all over the world, causing a new disease identified as COVID-19 [1,2]; then, on 12 March 2020, the World Health Organization (WHO) declared a global pandemic [3]. Variability in the susceptibility and severity at different ages has been observed since the beginning of the pandemic [4]. Children have been less frequently and severely affected than adults, requiring hospitalization only in 5–10% of cases [5,6,7,8]. Often, children affected by SARS-CoV-2 are asymptomatic or have mild symptoms, most commonly fever, a cough, pharyngitis, gastrointestinal symptoms and anosmia or hyposmia [5,9,10,11]. Although the COVID-19 disease course is generally mild in children, cases of severe infection have been described in a small proportion of patients; thus, therapeutic options such as dexamethasone, antivirals (e.g., remdesivir), convalescent plasma and monoclonal antibodies (mAbs) have been reported [12,13]. Nevertheless, experiences and data for each treatment are limited for the pediatric population. To date, three mAbs have been developed for mild-to-moderate COVID-19 infection and were approved between November 2020 and May 2021 by the Food and Drug Administration (FDA) with emergency use authorization (EUA). 

Casirivimab-imdevimab, also known as REGEN-COV, is an anti-spike mAb that has been authorized for the treatment of high-risk patients (>12 years of age, >40 kg) with mild-to-moderate COVID-19 [14]. This authorization was based on the results of a randomized placebo-controlled clinical trial enrolling 275 patients who reported a reduction in viral load [15]. Another retrospective cohort study enrolled 1392 patients and showed a reduction in hospitalization in patients who were treated with casirivimab-imdevimab compared to untreated controls [15]. 

Bamlanivimab, named LY-CoV555, is a mAb developed by a partnership between Ab Cellera Biologics and Eli Lilly [16]. This antibody was shown to be beneficial in the outpatient setting, reducing the hospital admission rate, and a randomized phase-two/three trial further showed a statistically significant reduction in the SARS-CoV-2 viral load, which was achieved faster than in patients who did not receive treatment [16]. Later, bamlanivimab was combined with etesevimab and additional trials showed a beneficial effect for accelerating the natural decline of the viral load over time [17]. Recently, in December 2021, the FDA approved the use of bamlanivimab-etesevimab for patients under 12 years old. 

Sotrovimab, formerly known as VIR-7831, is an engineered human mAb that neutralizes SARS-CoV-2 and multiple other sarbecoviruses [18]. The target of this mAb is a highly conserved epitope of sarbecoviruses, and the hypothesis behind its use is that this mAb can neutralize potential variants of SARS-CoV-2 [18]. Data from a multicenter, double-blind, phase-three trial reported that sotrovimab reduced the risk of disease progression [18]. 

Since the beginning of the pandemic, proliferation of SARS-CoV-2 variants has been observed with different levels of susceptibility to the mAbs available [19]. Therefore, there is a great need to find the therapeutic agents that alone or in combination can be effective against more variants of SARS-CoV-2 [19]. Today, limited data are available about the use of mAbs in children. Moreover, until now, data on mAb therapy in children have been limited to the pre-Omicron era and data on Sotrovimab are missing. 

In May 2021, a group of experts, based on the evidence, suggested that routine administration of mAb therapy for treatment of COVID-19 in children or adolescents was not permissible [20]. Yet, a recent report by Mak et al. showed a case-series of 17 patients with ages between 12 and 20 years old with COVID-19, who were treated with mAbs (bamlanivimab, bamlanivimab-etesevimab, casirivimab-imdevimab) [21] and the treatment was well-tolerated and safe, with no adverse reactions reported. To support this evidence, we report our experience of the administration of mAb as a treatment for COVID-19 in pediatric patients aged from 24 days to 18 years old. 

## 2. Materials and Methods

A retrospective study was conducted on patients who received mAb infusions for the treatment of mild-to-moderate COVID-19 at Bambino Gesù Children’s Hospital from April 2021 to January 2022. We applied AIFA’s indications for the use of anti-SARS-CoV-2 mAbs [22]: body mass index (BMI) ≥ 95th percentile for age and gender; chronic renal failure, including hemodialysis or peritoneal dialysis; uncontrolled diabetes mellitus (HbA1c > 9% or 75 mmol/mol) or chronic complications; primary and secondary immunodeficiencies; hemoglobinopathies; cerebral vascular diseases (including high blood pressure with organ damage); neurodevelopment and degenerative diseases; chronic obstructive pulmonary disease or other respiratory chronic conditions (for example, asthma, pulmonary fibrosis or condition requiring oxygen therapy not for SARS-CoV-2); chronic hepatopathy. Based on high-risk criteria reported by the FDA EUA, we included neonates and infants (age < 1 year) [23]. 

Monoclonal antibodies were administrated once we obtained a parent’s consent, and for patients younger than 12 years old, off-label mAb use was prescribed according to the hospital’s policy. Patients with symptoms that had occurred less than five days before were evaluated for treatment appropriateness either after referral by the primary care provider or from the emergency room after confirming Sars-CoV-2 infection through a reverse-transcriptase qualitative PCR assay of a nasopharyngeal swab. Recently, the identification of a variant of concern (VOC) of SARS-CoV-2 required samples be sent to the Clinical Virology Laboratory. Demographic and clinical data were extracted from the patients’ clinical records. The monoclonal antibody infusion doses (for children >12 years and ≥40 kg) and rates were as follows: Casirivimab (Roche, Monza, Italy) 1200 mg + Imdevimab (Roche, Monza, Italy) 1200 mg infused over 60 min, Bamlanivimab (Lilly Corporate Centre, Indianapolis, IN, USA) 700 mg + Etesevimab (Lilly Technology Center, Indianapolis, USA) 1400 mg infused over 30 min, Sotrovimab (GlaxoSmithKline S.p.A, Parma, Italy) 500 mg infused over 30 min. All patients were monitored for infusion-related reactions during and at least 60 min after infusion. For children with a weight under 40 kg or age below 12 years old, a reduced dose of each mAb was used according to anthropometric measurements. In detail, bamlanivimab-etesevimab was administered according to the FDA-approved schedule (ref); the casirivimab-imdevimab doses were 120 mg + 120 mg for weights 5 to <10 kg, 225 mg + 225 mg for weights ≥10 to <20 kg and 450 mg + 450 mg for weights ≥20 to <40 kg; the Sotrovimab doses were 125 mg for weights <20 kg and 250 for weights ≥20 to <40 kg. 

## 3. Results

A total of 73 pediatric patients (age 0–18 years) affected by COVID-19, with mild and moderate respiratory symptoms and with risk factors for severe disease, received monoclonal antibodies as their treatment. Of the study cohort, 46 patients received casirivimab-imdevimab (23 of them were <12 years), 22 children were treated with bamlanivimab-etesevimab (9 of them were <12 years) and four patients under 12 years old were treated with sotrovimab after confirmation of the Omicron variant (Table 1). The youngest patient was a neonate of 24 days of life treated with SARS-CoV-2 mAb for high fever and progressive lung worsening. The most common comorbidities in our cohort were secondary immunodeficiency and congenital heart disease (Table 1). All children were treated within five days of the COVID-19 diagnosis.

There were no significant adverse effects or reactions that required the cessation of the infusion such as anaphylaxis, hypotension or dyspnea. No one was admitted/readmitted for reasons related to COVID-19 or MIS-C within 30 days after receiving treatment. 

## 4. Discussion

The use of SARS-CoV-2 mAb may be a good therapeutic strategy for pediatric patients with COVID-19 at high risk of severe disease. The vaccination of children aged 5–11 years is still getting started in Italy, while increased transmissibility across all age groups and a higher incidence of infection for school-aged children have been reported [24]. Fin this context, vaccinating the youngest of children and the vaccine responses in immunocompromised populations are still debated [25]. Today, there are no approved clinical criteria for SARS-CoV-2 mAb administration under 12 years old and under 40 kg of body weight. Recently, the Italian Society of Pediatrics proposed pediatric criteria for the use of mAbs based on the available evidence from the scientific literature and experts’ recommendations [26]. Besides this, the American Academy of Pediatrics first, and the Minnesota Department of Health afterward, issued a list of criteria for SARS-CoV-2 mAb indications in pediatric populations [23]. These criteria overlapped with criteria used in adult populations, and we considered it appropriate to apply them to our study population. As a third-level children’s hospital receiving local referrals for pediatric SARS-CoV-2 infection, the majority of our patients were affected by a comorbidity. Our clinical decision to administer mAbs was mainly driven by the presence of one or more severe comorbidities. In this high-risk population, the administration of mAbs had the following goals: the prevention of severe COVID-19 and the prevention of a delay to their therapeutic course. As well as this, fast recovery from COVID-19 means a reduction of the harmful effects of persistent isolation of a hospital stay. 

Thus, we identified and essentially treated three categories of patients:

Children affected by primary or secondary immunodeficiency at high risk of chronic COVID-19 [27];
Children born with severe congenital heart disease at high risk of life-threatening cardiac complications [28];
Children with neurological or neurocognitive problems in which persistent isolation may worsen psychological/psychiatric aspects [29].

Furthermore, despite the lower morbidity and mortality in pediatric populations compared with adult patients, significant healthcare resources are still involved and COVID-19 infection among children may entail unknown long-term consequences.

The type of mAb was chosen based on drug availability, the epidemiology of SARS-CoV-2 variants of concern (VOCs) and laboratory VOC identification in the last month. Sotrovimab was administrated exclusively after SARS-CoV-2 VOC B.1.1.529 (Omicron) detection. 

Thus, prompt VOC identification by the laboratory is a mandatory tool to prescribe the appropriate mAb. 

Most of the patients (80%) received the SARS-CoV-2 mAb during hospitalization for infection; we admitted all high-risk patients to ensure clinical observation. 

To date, experience of administering antiviral drugs for COVID-19 treatment in pediatric populations is still limited, and therefore, sharing data about their efficacy and safety of SARS-CoV-2 mAbs might help improve clinical practice. However, based on the differing presentation of COVID-19 between adults and children, we cannot apply the adult efficacy outcomes to the pediatric population; specific criteria must instead be identified to design specific pediatric treatment practices.

These results should be interpreted with caution as there are significant limitations to this case-series, such as the lack of a control group. Moreover, the study was conducted mainly before the spread of the VOC Omicron, and therefore, the majority of patients received mAbs other than sotrovimab.

## 5. Conclusions

In conclusion, our experience suggests that mAbs against SARS-CoV-2 are safe and well-tolerated in pediatric populations at high risk of developing severe COVID-19 but more data are needed to confirm and further these results.

## Figures and Tables

**Table 1:** Use of monoclonal antibodies for age and comorbidities of our study group.

Type of mAb	*n* < 12 y	*n* > 12 y
Casirivimab-imdevimab	24	22
Bamlanivimab/etesevimab	9	14
Sotrovimab 500 mg	4	
*Total*	*37*	*36*
**Type of comorbidity**	***n***	
Secondary immunodeficiency	16	
Congenital heart disease	14	
Primary immunodeficiency	8	
Neurodevelopmental disorders	16	
Obesity	5	
Chronic boncopneumopathy	5	
Heart transplant	3	
Newborn	1	
Acquired heart disease	1	
Chronic kidney failure	1	
